# Complete Genome Sequence Reveals Evolutionary and Comparative Genomic Features of *Xanthomonas albilineans* Causing Sugarcane Leaf Scald

**DOI:** 10.3390/microorganisms8020182

**Published:** 2020-01-28

**Authors:** Hui-Li Zhang, Mbuya Sylvain Ntambo, Philippe C. Rott, Gongyou Chen, Li-Lan Chen, Mei-Ting Huang, San-Ji Gao

**Affiliations:** 1 National Engineering Research Center for Sugarcane, Fujian Agriculture and Forestry University, Fuzhou 350002, Fujian, China; hlzhang@fafu.edu.cn (H.-L.Z.); ntambos@africau.edu (M.S.N.); Lilan_Chen@163.com (L.-L.C.); hmt159379@163.com (M.-T.H.); 2 CIRAD, UMR BGPI, F-34398 Montpellier, France, and BGPI, Univ Montpellier, CIRAD, INRAE, Montpellier SupAgro, F-34398 Montpellier, France; philippe.rott@cirad.fr; 3 School of Agriculture and Biology/State Key Laboratory of Microbial Metabolism, Shanghai Jiao Tong University, Shanghai 200240, China; gyouchen@sjtu.edu.cn

**Keywords:** bacterial disease, *Xanthomonas albilineans*, average nucleotide identity (ANI), structural variation, specific genes

## Abstract

Leaf scald (caused by *Xanthomonas albilineans*) is an important bacterial disease affecting sugarcane in most sugarcane growing countries, including China. High genetic diversity exists among strains of *X. albilineans* from diverse geographic regions. To highlight the genomic features associated with *X. albilineans* from China, we sequenced the complete genome of a representative strain (Xa-FJ1) of this pathogen using the PacBio and Illumina platforms. The complete genome of strain Xa-FJ1 consists of a circular chromosome of 3,724,581 bp and a plasmid of 31,536 bp. Average nucleotide identity analysis revealed that Xa-FJ1 was closest to five strains from the French West Indies and the USA, particularly to the strain GPE PC73 from Guadeloupe. Comparative genomic analysis between Xa-FJ1 and GPE PC73 revealed prophage integration, homologous recombination, transposable elements, and a clustered regulatory interspaced short palindromic repeats (CRISPR) system that were linked with 16 insertions/deletions (InDels). Ten and 82 specific genes were found in Xa-FJ1 and GPE PC73, respectively, and some of these genes were subjected to phage-related proteins, zona occludens toxin, and DNA methyltransferases. Our findings highlight intra-species genetic variability of the leaf scald pathogen and provide additional genomic resources to investigate its fitness and virulence.

## 1. Introduction

*Xanthomonas* is a genus in the gamma subdivision of the Proteobacteria that contains a large number of plant pathogens. Members of the genus cause disease on at least 124 monocots and 268 dicots and provide excellent case studies for the understanding of molecular plant-microbe interactions [1]. Leaf scald caused by *X. albilineans* is an important disease that can have considerable economic impact on sugarcane industries worldwide [2].

*X. albilineans* colonizes the vascular system of sugarcane leaves and stalks, but is also capable of infecting the parenchyma cells of sugarcane, a unique characteristic differing from other bacterial pathogens with a reduced genome [3]. This bacterial pathogen induces various leaf and stalk symptoms during disease progress [2]. In the initial phase of the disease, *X. albilineans* causes the appearance of white, narrow, sharply defined leaf stripes which is followed by necrosis and wilting of infected leaves, thus resulting in plant death [4,5]. In mature and diseased stalks, side shoots develop along the stalk from the node buds and basal side shoots are always more developed than those higher up [2,4]. *X. albilineans* produces the toxin albicidin, which has phytotoxic and antibiotic properties. Albicidin is a potent DNA gyrase inhibitor that inhibits chloroplast DNA replication and blocks chloroplast differentiation, thus resulting in the white stripe and chlorotic symptoms on infected leaves [6].

A high genetic diversity has been reported among worldwide strains of *X. albilineans* that are currently distributed in three serovars and six lysovars. Serovar I includes isolates from Australia, Mauritius, South Africa, Guadeloupe, India, and the United States. Serovar II consists of isolates from African countries, and serovar III contains isolates from the Caribbean Islands (Martinique, Guadeloupe, Saint-Kitts), Sri Lanka, and Fiji [7,8]. The serological variability of *X. albilineans* has been confirmed using a combination of monoclonal antibodies and DNA fingerprinting with 38 strains of the pathogen from various geographical locations [9]. At least 10 genetic groups (PFGE-A to PFGE-J) have been described using pulsed-field gel electrophoresis and by multi-locus sequence analysis (MLSA) [10,11]. However, our previous studies showed that the genetic diversity of *X. albilineans* from China is very low. MLSA analysis of 14 strains from this country revealed that they belong to the PFGE group B, based on five housekeeping genes [12,13]. With the advent of sequencing technologies, genome sequence data can help to resolve the phylogeny among the strains of *X. albilineans* by considering mutation and recombination at the whole-genome level [14].

The complete genome sequence has been determined for several *Xanthomonas* species, making these bacteria attractive models to study plant-pathogen interactions at the molecular level. Comparative studies have also improved our understanding of the genome features of various bacterial pathogens. For example, the full genome sequence of *X. albilineans* strain GPE PC73 from Guadeloupe lacks the *hrp* genes encoding a type III secretion system (T3SS) present in almost all gram negative plant pathogenic bacteria [11,15]. Moreover, *X. albilineans* experienced a reduced genome evolution in comparison to other sequenced plant pathogenic xanthomonads. This bacterial species is phylogenetically close to *Xylella fastidiosa*, another xylem-invading pathogen with a reduced genome and that is also missing an *hrp* T3SS [15,16]. Phylogenetic analyses with rRNA sequences excluded *X. fastidiosa* from the *Xanthomonas* group, but phylogenetic analysis with *X. albilineans* genomic sequences suggested that *X. fastidiosa* belongs to the *Xanthomonas* group [15].

So far, genome sequencing has been reported for 15 worldwide strains of *X. albilineans*, including 14 strains with scaffolds, using the Illumina technique, and one strain (GPE PC73) using the Sanger technique [11,15]. This genomic information appears to be very useful to explore the biological characteristics of *X. albilineans*, the reductive genome features, and the intra-species genetic diversity of this pathogen. The aforementioned strains did not include any strain from China, where several outbreaks of sugarcane leaf scald were recently reported [12,17,18]. Therefore, to better understand the genome features of *X. albilineans* between China and other counties, we generated the complete genome without gaps of a representative strain from China (Xa-FJ1) [12] using the PacBio RSII and Illumina HiSeq PE150 platforms. The genome of this strain was compared to the genome of the other sequenced worldwide strains of *X. albilineans*, particularly with the complete genome of strain GPE PC73 from Guadeloupe.

## 2. Materials and Methods

### 2.1. Isolation of Bacteria and DNA Preparation

*X. albilineans* strain Xa-FJ1 was isolated from a leaf section originating from a diseased sugarcane plant of clone YG48 collected at Zhangzhou, Fujian Province, China [12]. A pure culture of this strain was grown with constant shaking at 200 rpm and 28 °C for 48 h in XAS liquid medium [19]. Bacterial genomic DNA was extracted from cultures of Xa-FJ1 using the SDS method [20]. Extracted genomic DNA was subjected to quality control by agarose gel electrophoresis and quantified using the Qubit v.2.0 fluorometer (Life Technologies, Carlsbad, CA, USA).

### 2.2. Genome Sequencing and Assembly

Whole-genome sequencing was performed using the PacBio RSII platform [21] and Illumina Hiseq platform at Beijing Novogene Bioinformatics Technology Co., Ltd. A 10-Kb DNA library was constructed using the SMRT bell TM Template kit v.1.0, according to the manufacturer’s instructions, and sequenced using single-molecule real-time (SMRT) sequencing technology. A 350 bp sequencing library was prepared with NEBNext^®^ Ultra™ DNA Library Prep Kit for Illumina (New England Biolabs (Beijing) LTD, Beijing, China) in accordance with the manufacturer’s recommendations, and sequenced on Illumina Hiseq platform with a 2 × 150 bp paired-end sequencing kit. After quality control of the sequencing data, clean reads from the PacBio platform were assembled de novo with the SMRT Link v.5.0.1 software (Pacific Biosciences, Menlo Park, CA, USA) using the hierarchical genome assembly process (HGAP) [22]. The assembly results were further corrected with Illumina data using the bwa software (https://sourceforge.net/projects/bio-bwa/). The complete sequence of the genome of *X. albilineans* strain Xa-FJ1 has been deposited at GenBank under the accession number CP046570-CP046571.

### 2.3. Genome Component Prediction and Gene Annotation

Putative open reading frames (ORF) were predicted using the GeneMarkS v.4.17 program [23] (http://topaz.gatech.edu/GeneMark/). Transfer RNA (tRNA) genes were predicted with tRNAscan-SE [24]. Ribosome RNA (rRNA) genes were analyzed using rRNAmmer [25]. Small RNAs (sRNA) were predicted by BLAST against the Rfam database [26,27], and confirmed using the cmsearch program (version 1.1rc4) with default parameters. Gene annotations were determined with the BLASTP program (E-value < 1 ×10^−5^, identity ≥ 40%, coverage ≥ 40%) and six databases including GO (gene ontology) [28], KEGG (Kyoto encyclopedia of genes and genomes) [29,30], COG (clusters of orthologous groups) [31], NR (non-redundant protein databases) [32], TCDB (transporter classification database) [33], and Swiss-Prot [34]. Genome overview was created by Circos to show the annotation information [35].

The secretory proteins were predicted with SignalP (version 4.1, http://www.cbs.dtu.dk/services/SignalP-4.1/) [36] and TMHMM (Version 2.0c, http://www.mybiosoftware.com/tmhmm-2-0c-prediction-transmembrane-helices-proteins.html). Since *X. albilineans* is a bacterial pathogen, pathogenicity and drug resistance data were also retrieved from the pathogen–host interactions database (PHI-base) [37], the virulence factors of pathogenic bacteria database (VFDB) [38], and the antibiotic resistance genes database (ARDB) [39]. Carbohydrate-active enzymes were predicted using the carbohydrate-active enzymes database [40]. CRISPRs were identified using CRISPRdigger (version 1.0, https://github.com/greyspring/CRISPRdigger) [41].

### 2.4. Average Nucleotide Identity and Phylogenetic Analysis

The genome sequences of 15 worldwide strains of *X. albilineans* and one strain of *Xanthomonas pseudalbilineans* (used as outgroup) were retrieved from NCBI (Table S1). The average nucleotide identities of these 16 strains and strain Xa-FJ1 sequenced in this study were calculated by pairwise genome comparison based on BLAST+ with JSpeciesWS (http://jspecies.ribohost.com/jspeciesws/) [42,43]. Core genes and specific genes of the 16 strains of *X. albilineans* were analyzed using the CD-HIT rapid clustering of similar proteins software with a threshold of 50% pairwise identity, and 0.7 length difference cut off of amino acids [32,44,45]. Gene sequences were aligned pairwise and sequence redundancies were removed using the BLAST option of the solar software [46]. The gene family clustering was based on the alignment results using Hcluster-sg software (https://github.com/douglasgscofield/hcluster). A phylogenetic tree, based on 2341 core genes conserved across the 16 strains of *X. albilineans*, was constructed using the neighbor-joining method and 1000 bootstrap replications with TreeBeST (http://treesoft.sourceforge.net/treebest.shtml).

### 2.5. Comparative Genomic Analysis

Chromosome alignment between strains Xa-FJ1 and GPE PC73 of *X. albilineans* was performed using MUMmer version 3.22 [47] and LastZ version 1.02.00 tools [48,49]. Scattered comparison results were linked into longer forms by the chainNet package. Structural variations (SV) like translocation, inversion and trans + inverse relationships in the comparison blocks were identified according to arrange relationships and relative orientations. The results were visualized using Circos (http://circos.ca/) [35].

## 3. Results

### 3.1. General Genomic Features of X. albilineans Strain Xa-FJ1

A total of 775 Mb PacBio clean data (N50 read length of 15,957 bp, quality of 0.84; average read length of 10,889 bp) was generated with an estimated 206× average depth of sequencing coverage. Preliminary assembly was conducted with SMRT Link v.5.0.1 and corrected by the variant Caller module. The assembly result was further corrected with 2141 Mb Illumina clean data (89.99% of bases with quality score >30) using bwa. This genome was assembled into one circular chromosome of 3,724,581 bp (Figure S1A) and one single plasmid of 31,536 bp (Figure S1B). This chromosome contained 3176 predicted genes with an average gene length of 1016 bp. The genome of strain GPE PC73—the only other *X. albilineans* strain with a complete genome sequence in NCBI—contained 3115 putative genes with an average length of 1059 bp (Table 1). Besides the circular chromosome, one plasmid was present in strain Xa-FJ1 but strain GPE PC73 had three plasmids. The nucleotide sequence of the plasmid of strain Xa-FJ1 shared 99.91% identity with PlasmII of strain GPE PC73 (Table S2).

### 3.2. Functional Annotation of the Predicted Genes of X. albilineans Strain Xa-FJ1

Gene annotation was performed with 11 different databases (Table S3). Genes involved in metabolism pathways were significantly enriched based on GO, KEGG, and COG databases. Among the 24 subcategories of biological processes of the GO database, the largest category of Xa-FJ1 was assigned to metabolic process (1186 genes) (Figure S2). Using KEGG annotation, 1491 of 2987 annotated genes were involved in metabolism, especially the metabolic pathways belonging to global and overview maps (529 genes) (Figure S3). Amino acid transport and metabolism was ranked the third largest category among the 25 classes of functional categories of the COG database (Figure S4). A total of 279 putative virulence-associated factors were identified based on the VFDB database, including 53 flagella-related genes, 27 genes related to type IV pili, and 35 genes related to different secretion systems (Table S4).

**Figure 1:**
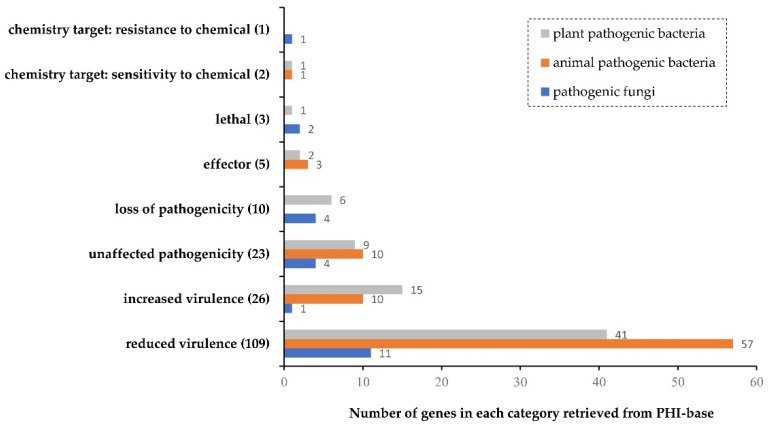
Phenotype classification of putative pathogenicity genes of *X. albilineans* retrieved from the pathogen–host interactions database (PHI-base).

Genes potentially involved in pathogenicity of Xa-FJ1 were also identified by blasting the pathogen–host interactions database (PHI-base) (Figure 1 and Table S3). Among the 179 genes with homologous sequences in the PHI-base, 23 shared homologs with infection-related genes in pathogenic fungi such as *Magnaporthe oryzae*. Eighty-one and 75 genes were homologous to virulence factors characterized in bacteria pathogenic to animals and plants, respectively. Fifty three of the 75 bacterial virulence factors with hit in Xa-FJ1 were from *Xanthomonas* spp. Six Xa-FJ1 genes, homologous to sequences essential for full virulence in plant pathogenic bacteria, were XaFJ1_GM001161 (*rsmA* in *X. oryzae*), XaFJ1_GM001582 (*galU* in *X. campestris*), XaFJ1_GM001983 (*pstB* in *X. citri*), XaFJ1_GM002139 (*rpoN* in *Erwinia amylovora*), XaFJ1_GM002197 (*vrpA* in *X. citri*), and XaFJ1_GM002596 (*hrpM* in *X. citri*).

In particular, the quorum sensing (QS) signal molecule Ax21 of *X. oryzae* pv. *oryzae* was retrieved from the PHI-base. Thirteen genes belonging to four operons (*raxSTAB*, *raxPQ*, *raxRH* and *phoPQ*) that are supposed to be required for the well-known effector Ax21 activity in *X. oryzae* pv. *oryzae* were also identified in *X. albilineans* strain Xa-FJ1 (Table S3). Four copies of *raxB* (XaFJ1_GM001779, XaFJ1_GM001890, XaFJ1_GM001891, XaFJ1_GM002569) were included, but *raxA* and *raxST* were not retrieved from the PHI-base. XaFJ1_GM000725 was homologous to gene *raxC* outside the *raxSTAB* locus in *X. oryzae* pv. *oryzae*, and may encode the outer membrane component of the type I secretion system. The retrieved genes of Xa-FJ1 also contained three copies of *raxR* (XaFJ1_GM000629, XaFJ1_GM000871, XaFJ1_GM002789) and one *raxH* ortholog (XaFJ1_GM000872), which form two-component regulatory systems. XaFJ1_GM000222 and XaFJ1_GM000223 were related to the operon *phoP*/*phoQ* of *X. oryzae*, which is involved in reduction and increase of virulence. XaFJ1_GM002301 and XaFJ1_GM002300 were present in an operon that corresponds to the operon *raxP*/*raxQ* of *X. oryzae*, which is involved in Ax21 tyrosine sulfation.

### 3.3. Average Nucleotide Identity and Phylogenetic Analysis among Strains of X. albilineans

**Figure 2:**
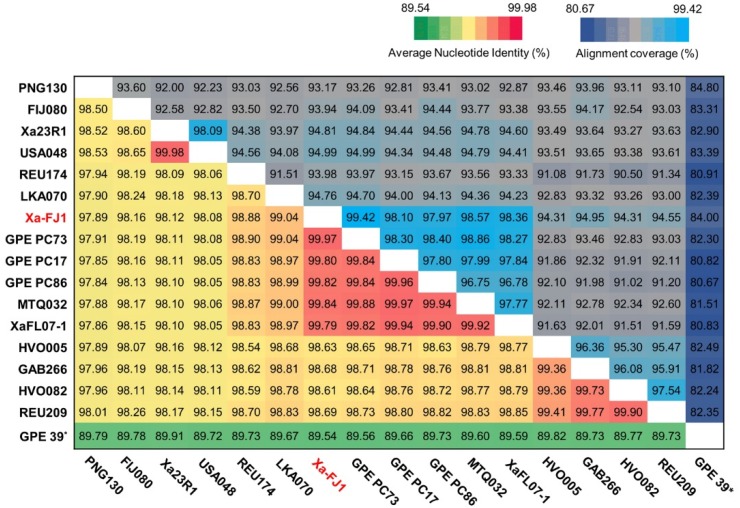
Heat map of average nucleotide identity (ANI) and alignment coverage based on the entire genome sequence of 16 strains of *X. albilineans* and one strain of *X. pseudalbilineans*. Strain Xa-FJ1 sequenced in this study is written in red. ANI values (%) and alignment coverage (%) of each two-genome sequence comparison are shown in the lower triangle and in the upper triangle of the matrix, respectively. * The last bacterial strain (GPE 39) does not belong to *X. albilineans* but to *X. pseudalbilineans* and was used as an outgroup.

Average nucleotide identity (ANI) analysis was conducted with the full-length genome sequence of Xa-FJ1 and 15 other strains of *X. albilineans* and one strain of *X. pseudalbilineans* (GPE 39) (Figure 2 and Table S1). ANI varied from 97.84–99.98%% among the 16 worldwide strains of *X. albilineans*. These strains shared 89.54–89.91% ANI with strain GPE 39 of *X. pseudalbilineans*. Strain Xa-FJ1 from China had 97.89–99.97% ANI with the 15 other strains of *X. albilineans* in the NCBI database. Strain Xa-FJ1 had the highest identity (≥99.79%) with four strains from the French West Indies (GPE PC73, GPE PC17, GPE PC86 from Guadeloupe and MTQ032 from Martinique), and one strain from the USA (XaFL07-1).

**Figure 3:**
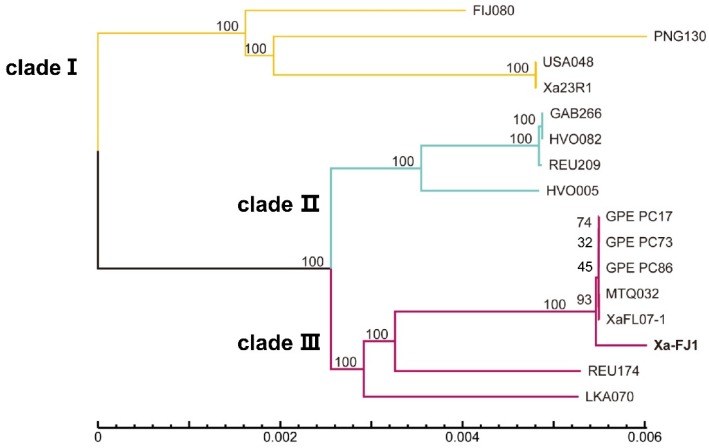
Neighbor-joining phylogenetic tree of *X. albilineans* constructed with the core-genome sequence of 16 strains of *X. albilineans* using TreeBesT software. Bootstrap values based on 1000 replications are indicated at branches. Scale bar units are in number of substitutions per nucleotide.

The 16 strains of *X. albilineans* were distributed into three distinct clades of a phylogenetic tree based on the core-genome (2341 genes) of these strains. Strain Xa-FJ1 from China was assigned to a specific branch or sub-group of clade III that also included four strains from the French West Indies (GPE PC73, GPE PC17 and GPE PC86 from Guadeloupe, and MTQ032 from Martinique) and one strain from the USA (XaFL07-1) (Figure 3). Strain REU174 from Reunion Island, and strain LKA070 from Sri Lanka, were also assigned to clade III but were located in two other sub-groups. Clade II was formed by three strains from Africa (GAB266, HVO082, and HVO005) and one strain, REU209, from Reunion Island, an island off the coast of East Africa. Clade I contained four strains: One from Fiji (FJI080), one from Papua New Guinea (PNG130), and two from the USA (USA048 and Xa23R1).

### 3.4. Chromosomal Structural Variation Between Strains Xa-FJ1 and GPE PC73 of X. albilineans

**Figure 4:**
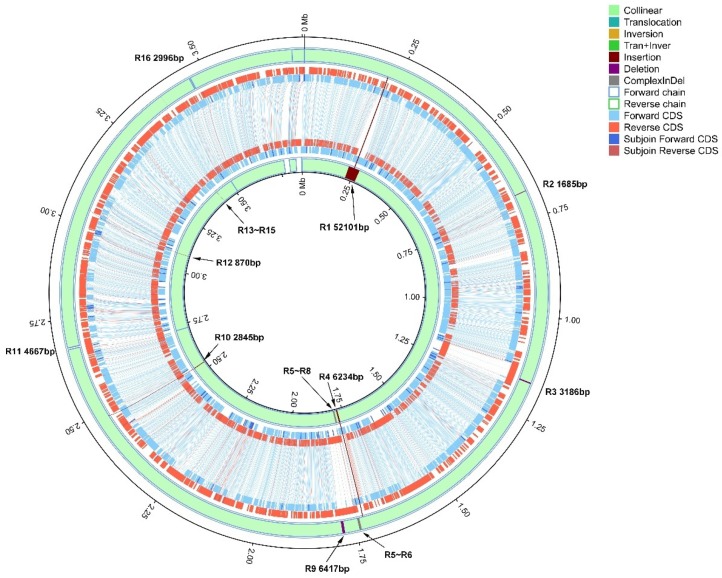
Chromosomal structural variations between strains Xa-FJ1 and GPE PC73 of *X. albilineans*. The chromosomal sequence of strain Xa-FJ1 was designated as the reference. Moving inwards, the first three circles show protein coding sequences (CDSs) conserved in Xa-FJ1, and the inner three circles show CDSs conserved in GPE PC73. The grey lines between the predicted CDSs indicate consistent alignment.

GPE PC73 was one of the closest strains to Xa-FJ1 and, as mentioned above, was the only *X. albilineans* strain with a complete genome sequence in the NCBI. Comparative analysis of the chromosomes between Xa-FJ1 and GPE PC73 identified 16 structural variations that were linked to various insertion/deletions (InDels, R1~R16) (Figure 4 and Table 2). In comparison to strain Xa-FJ1, the chromosome of strain GPE PC73 contained an insertion from position 219,691 bp to 271,791 bp (52,101 bp in R1 region). This region included 72 specific genes in the genome of strain GPE PC73 that were absent in strain Xa-FJ1 (Table 2 and Table S5). A plasticity zone (about 7 Kb long) of the chromosome of strains Xa-FJ1 and GPE PC73 had low similarity (40.26%) between the two strains and consisted of four InDels (R5, R6, R7, and R8).

Five InDel fragments were flanked by repetitive sequences. Three of them (R3, R4 and R9) were expected to be involved in synthesis of the AMP-binding super family domain of a non-ribosomal peptide synthase. The other two InDel fragments were located at position 679,518 bp to 681,202 bp (R2) in strain Xa-FJ1, and 2,532,506 bp to 2,535,350 bp (R10) in strain GPE PC73, and were predicted to encode hypothetical proteins and a putative methyl-accepting chemotaxis protein, respectively.

InDel fragments at position R11 and R16 contained XaFJ1_GM002292 and XaFJ1_GM002989, respectively, and are expected to encode putative transposases. Homologous sequences occurred on both sides of XaFJ1_GM002292 and XaFJ1_GM002989, resulting in the similarity of adjacent genes. In contrast, no homologous sequence was found on either side of insertion fragment R12 at loci XALc_2603 and XALc_2604 of strain GPE PC73, although these two genes were also associated to transposases. In addition, R12 had the same sequence as loci XALc_1241-XALc_1245 that were associated to replicative transposition. A sequence variation at position 3.4 Mb (R13-R15) between strains Xa-FJ1 and GPE PC73 included clustered regulatory interspaced short palindromic repeats (CRISPR), downstream of CRISPR-associated protein 2 (*cas2* gene).

Like the genome of GPE PC73, the genome of strain Xa-FJ1 contained two CRISPR/cas systems: CRISPR-1 and CRISPR-2. The CRISPR-2 system was 100% identical between the two strains and the CRISPR-1 system shared 76.77% identity between Xa-FJ1 and GPE PC73 (Figure S5). The CRISPR-1 system of GPE PC73 is associated with seven *cas* genes and contains thirty-four 31-base pair repeats and thirty-three 33- to 38-base pair spacers [11]. The nucleotide sequence of the three spacers of the trailer end (33, 32, 31) varied between strains Xa-FJ1 and GPE PC73. Genome variations R13-R15 between the two strains corresponded to seven spacers of CRISPR-1 that were present in only one of the two strains (Figure S5).

### 3.5. Chromosomal Specific Genes between Strain Xa-FJ1 and Strain GPE PC73 of X. albilineans

Of the 3176 putative protein-coding sequences (CDSs) manually annotated on the chromosome of *X. albilineans* strain Xa-FJ1, 2998 hit in the CDSs of strain GPE PC73, and another 178 hit in the chromosomal sequence of strain GPE PC73, which were not annotated as CDSs due to different prediction methods. Only 10 predicted CDSs were specific to strain Xa-FJ1 from China (Table 3), nine of which (XaFJ1_GM001517~XaFJ1_GM001525) were located in the plasticity zone (R5-R8, Figure 4 and Table 3). Six genes were associated with hypothetical proteins. XaFJ1_GM001517 was predicted to encode a protein involved in synthesis of the zona occludens toxin (Zot). XaFJ1_GM001523 was expected to code for a DNA-binding protein homologous to the one from *Xanthomonas* phage phiLf. The protein encoded by XaFJ1_GM001524 was similar to the replication initiation protein from *X. translucens*. Thus, these genes might be acquired as prophage. The last specific gene (XaFJ1_GM002989) was located far away from the plasticity zone, and was predicted to encode a transposase with an ATP-binging function (R16).

A total of 138 putative CDSs present in the genome of strain GPE PC73 (82 on the chromosome and 56 on two plasmids) were not found in the genome of strain Xa-FJ1. Strain GPE PC73 had an insertion region of 52 kb (R1, Figure 4) that contained 72 genes specific to GPE PC73 (Table S5). Among these, 53 genes had no predicted function and were considered hypothetical proteins (including one hypothetical secreted protein). Fifteen genes were annotated as hypothetical phage-related proteins, including XALc_0206 (hypothetical phage terminase large subunit protein) and XALc_0242 (putative phage integrase protein). The other four genes in the R1 region were predicted to be two putative N6 adenine-specific DNA methyltransferase proteins (XALc_0178 and XALc_0231), one putative DNA (cytosine-5-)-methyltransferase protein (XALc_0203), and one hypothetical DNA methyltransferase protein (XALc_0202). The 10 specific genes in the plasticity zone of GPE PC73 (R5-R8) included genes predicted to code for three hypothetical phage-related proteins (XALc_1539, XALc_1543 and XALc_1544), one putative filamentous phage cf1c related protein (XALc_1537), and one putative F pilin acetylation protein (XALc_1536).

### 3.6. Genes Present in Plasmids, PlasmI and PlasmIII, of Strain GPE PC73 of X. albilineans from Guadeloupe and Absent in Strain Xa-FJ1 from China

The plasmid common to strain GPE PC73 (PlasmII) and strain Xa-FJ1 (pXaFJ1) shared 99.91% identity between the two strains, and differed by 29 single nucleotide polymorphisms (SNPs). Two plasmids (PlasmI and PlasmIII) present in strain GPE PC73 were not found in Xa-FJ1. These two plasmids were predicted by the NCBI Prokaryotic Genome Annotation Pipeline to encode 24 and 32 genes, respectively (Table S6). As also reported by Pieretti et al. [11], PlasmI and PlasmIII each harbor an incomplete conjugal transfer system, referring to 12 genes and 11 genes, respectively. This system is considered as an important bacterial factor, helping bacterial adaptation to new hosts. Other genes of these two plasmids are predicted to code for proteins involved in replication and maintenance of plasmids such as plasmid partitioning proteins (XALp_3174, XALr_3236, and XALr_3237), plasmid stabilization system proteins (XALp_3179, XALp_3197, and XALr_3241) and the trans-acting replication initiator TrfA protein (XALr_3265). XALp_3176 of PlasmI has been annotated as a probable fic-family protein involved in adenylation of Rho-family GTPases. In PlasmIII, XALr_3238 is predicted to be a zinc metalloproteinase with a sprT domain that is a regulator of the *bolA* gene during the stationary phase. PlasmIII also harbors three genes (XALr_3245, XALr_3246, and XALr_3266) that putatively code for transcription regulator proteins.

## 4. Discussion

In recent years, leaf scald of sugarcane has been reported with increasing frequency in five sugarcane-growing regions of China by PCR-based detection [12,17,18]. Very low genetic diversity was found among strains of *X. albilineans* from China, based on sequence analysis of an ABC transporter gene (XALc_1791) and MLSA, suggesting recent spread in this country of a single strain (from genetic group PFGE-B) of the leaf scald pathogen [12,13]. Since 2009, 15 genome sequences of *X. albilineans* strains from 10 worldwide geographical locations have been deposited in GenBank with complete or scaffold sequences. Some genomic information is, however, not accessible for most of these strains because of incomplete sequences. No complete genome sequence of *X. albilineans* from China had been reported until now. Third-generation sequencing technology such as the PacBio system can help to resolve most of the assembly problems by providing long reads, low degree of bias and epigenetic classification [50]. Furthermore, this sequencing platform is competent to determine sequences with tandem repeats, high/low G + C values, and interspersed repeated regions, compared to second-generation sequencing platforms [50,51]. In this study, the complete genome of a representative strain of *X. albilineans* from China (Xa-FJ1) was sequenced without gaps using the PacBio RSII technique and was corrected by Illumina data for higher consensus accuracy.

The annotations performed with the GO, KEGG and COG databases indicated that a wide variety of metabolic related proteins were encoded by *X. albilineans* strain Xa-FJ1. These proteins included putative pectinesterase (XaFJ1_GM001902), cellulase (XaFJ1_GM000462) and xylose isomerase (XaFJ1_GM000104), and they were expected to be involved in active metabolic pathways for specific adaptation of this pathogen to the nutrient-poor xylem vessels. Among the 279 genes with homologous sequences in the VFDB database (Table S4), 8 genes have been shown to affect pathogenicity of the causal agent of sugarcane leaf scald by transposon mutagenesis [52]. The pathogen–host interactions database (PHI-base) collects gene phenotype and function data obtained by manual curation of the peer-reviewed literature [37]. Among 179 genes retrieved from PHI-base, 39 genes had putative functions previously reported [11,52]. Moreover, 20 of these genes referred to two-component systems. Among the remaining 140 genes, 98 were associated with reduced virulence or loss of pathogenicity and 5 genes were annotated as effectors, including Ax21. As a quorum sensing signal molecule in *X. oryzae*, Ax21 regulates cell density-dependent expression of up to 489 genes [53]. Thirteen genes involving four operons (*raxSTAB*, *raxPQ*, *raxRH* and *phoPQ*) were also identified in PHI-base, and these genes were associated with the activity of Ax21 in *X. oryzae.* Knockout mutants of these genes increased virulence (hypervirulence) to rice, except for *phoP*. Mutants of *raxST*, *raxA*, *raxB* or *raxC* from *X. oryzae* can evade XA21-mediated immunity to different degrees and induce disease lesions on rice expressing the XA21 receptor [54]. Both *raxH*/*raxR* and *phoP*/*phoQ* serve as two-component regulatory systems which may sense Ax21 and activate downstream signaling [53]. XaFJ1_GM002301 and XaFJ1_GM002300 from Xa-FJ1 are two genes corresponding to the operon *raxP*/*raxQ* of *X. oryzae* which is indispensable for the biological activity of Ax21 in the rice bacterial blight pathogen [55]. The functions of Ax21 and Ax21-related *rax* genes in Xa-FJ1 need to be explored to determine their role in the virulence of *X. albilineans*.

Based on complete sequence analysis, strain Xa-FJ1 from China shared high ANI with five strains of *X. albilineans* from the French West Indies (including GPE PC73 from Guadeloupe) and the USA (Florida). These six strains also clustered together in a phylogenetic tree based on the core-genome sequence of 16 strains of the pathogen. These results are congruent with those obtained in a previous study based on an ABC transporter gene (XALc_1791) and five housekeeping genes [13], suggesting a common evolutionary route for these six strains of *X. albilineans*. We hypothesized that the occurrence of *X. albilineans* in mainland China is linked to former frequent introductions of sugarcane germplasm and cultivars from Taiwan and foreign countries such as the USA, India, and Australia. These introductions occurred in order to enhance the local genetic diversity of parental clones in breeding programs during the 1980–1990s. Import of infected material has most likely occurred because *X. albilineans* can be present in cuttings collected from asymptomatic sugarcane, and the quarantine procedures used in China prior to 1990 were not as robust as they are nowadays [12].

Virulence of *X. albilineans* strains (data from the literature detailed in Table S1) was not correlated with distribution of the strains by ANI analysis. Strains with high ANI values showed great variation in virulence, suggesting that small genome differences (such as point mutations or small indels) were involved in variations of the virulence of *X. albilineans*. Additional virulence data are needed to further investigate the pathogenicity of *X. albilineans* and its relationship with adaption and evolution of this pathogen.

Chromosomal structural variations between strains Xa-FJ1 and GPE PC73 suggested that homologous recombination and horizontal gene transfer were tightly associated to genome evolution of *X. albilineans*. Five variable regions (R2, R3, R4, R9 and R10, Figure 4 and Table 2) appear to be caused by homologous recombination because the sequences flanking the InDel sites are identical in both bacterial strains, and homologous recombination may have occurred during chromosome replication. Alternatively, these regions may have resulted from assembly errors of GPE PC73 because the five InDels have no specific meaning as they only create internal duplicated DNA regions [56]. Horizontal gene transfer (HGT) is a common process responsible in prokaryotes for movement of genes from one organism to another, including antibiotic resistance genes and virulence factors [57]. Based on genome analysis of multiple strains of a given bacterial species or different species, prophages are the main cause of bacteria’s short-term intraspecies or interspecies diversity [58]. In this study, two prophage integrations (R1 and R5-R8) resulted in the addition of different specific genes in the genomes of strains Xa-FJ1 and GPE PC73. Regions R1 and R5–8, differing between Xa-FJ1 and GPE PC73, contain four DNA methyltransferases and an F pilin acetylation protein in strain GPE PC73. Various lytic and lysogenic phages have been proven to encode multi- and mono-specific orphan methyltransferases that have the ability to confer protection from restriction endonucleases of their bacterial hosts [59]. Gene XaFJ1_GM001517 of strain Xa-FJ1 (which is absent in GPE PC73) is predicted to encode the Zot protein (PD0928), which plays a role in pathogenicity of *X. fastidiosa*, suggesting a selective advantage for strain Xa-FJ1 from China [60]. These proteins may contribute to variation in virulence of *X. albilineans* or adaptation to certain sugarcane cultivars.

Strains Xa-FJ1 and GPE PC73 of *X. albilineans* have a smaller chromosome size (3.8 Mb) than those of other *Xanthomonas* species sequenced so far (generally about 5 Mb). *X. albilineans* does not possess a Hrp-type III secretion system, indicating that the pathogenicity of this pathogen must rely on other virulence factors or secretory systems [11]. Furthermore, *X. albilineans* possesses a T3SS system of the SPI-1 (for Salmonella Pathogenicity Island-1) injectisome family, which strongly suggests that this bacterial species also interacts with an unknown insect [15,61]. Zot protein acts like a physiological modulator that is used by animal pathogens, such as *Vibrio cholerae* and *Neisseria meningitidis*, to induce a reversible opening of tight junctions between cells and to increase the paracellular permeability in a non-toxic manner [62]. Presence of this gene is further evidence of a possible association of *X. albilineans* with an animal host that remains to be identified.

Transposable elements can also promote genome plasticity in bacterial genomes [63]. Three regions (R11, R12 and R16) of the *X. albilineans* genome contained transposases that were different between Xa-FJ1 and GPE PC73. The two strains also differed by their CRISPR-Cas signatures. The CRISPR-Cas system is a prokaryotic immune system among bacteria and archaea that provides resistance to foreign genetic elements [64]. Although strains Xa-FJ1 and GPE PC73 showed high ANI, these genomic features suggested rapid DNA adaptation of these trains during their evolution in different environments.

Many *Xanthomonas* strains also carry extra-chromosomal circular DNA in the form of plasmids, which can offer a myriad of benefits to the bacterial host, like virulence traits and antibiotic resistance [65]. Although strains Xa-FJ1 from China, and GPE PC73 from Guadeloupe had the closest evolutionary relationship, PlasmI and PlasmIII from GPE PC73 were absent in Xa-FJ1, thus suggesting that these DNA elements are not critical for basic virulence of the leaf scald pathogen, but might be essential for adaptation of *X. albilineans* to certain environments. The Fic (filamentation induced by cyclic AMP) domains are conserved from bacteria to humans. Fic domain proteins catalyze the addition of AMP, or ‘adenylylation’, of target host proteins [66]. For example, the secreted antigen IbpA from *Histophilus somni* causes collapse of the host-cell actin cytoskeleton by AMPylation to modify the three mammalian Rho family GTPases [67]. It remains to be determined whether XALp_3176 in PlasmI of GPE PC73 also assists *X. albilineans* to target the Rho-family GTPases in sugarcane. Lytic transglycosylases are bacterial enzymes that catalyze the non-hydrolytic cleavage of the peptidoglycan structures of the bacterial cell wall [68]. They are ubiquitous in bacteria which take part in a series of astonishingly diverse biological processes, such as cell wall metabolism, detection of cell-wall-acting antibiotics, insertion of secretion systems and flagellar assemblies into the cell wall and pathogenesis of certain gram-negative bacteria [69]. As a probable lytic transglycosylase protein, XALr_3249 in PlasmIII could be an attractive new target for the study of cell wall and virulence of *X. albilineans* in specific hosts. The importance of PlasmII shared by both strains also remains to be investigated.

## 5. Conclusions

We reported the first complete genome of a strain of *X. albilineans* (Xa-FJ1) from China using the PacBio RSII and Illumina HiSeq platforms. ANI analysis revealed that strain Xa-FJ1 had the closest evolutionary relationships with five strains of the pathogen from the French West Indies and the USA (Florida), particularly with strain GPE PC73 from Guadeloupe. These strains of *X. albilineans* have been associated with the most recently reported outbreaks and/or aerial spread of leaf scald in China, Florida, several Caribbean Islands, and Cuba. Genome comparison analysis suggested that horizontal gene transfer and homologous recombination were tightly linked to genome evolution of *X. albilineans*. Our findings contribute additional genomic resources to further investigate the diversity and pathogenicity of the causal agent of sugarcane leaf scald.

## Figures and Tables

**Table 1:** General chromosomal features of *X. albilineans* strains Xa-FJ1 from China and GPE PC73 from Guadeloupe.

Element and Characteristics	Xa-FJ1	GPE PC73
Sequencing platform	PacBio RSII, Illumina PE150	Sanger
Coverage	206×, 570×	17×
Size (bp)	3,724,581	3,768,695
G + C content (%)	63	63
No. protein-coding sequences (CDSs)	3176	3115
Coding density (%)	86.66	84
Average length in bp of all CDSs	1016	1059

**Table 2:** Chromosomal structural variation between *X. albilineans* strains Xa-FJ1 from China and GPE PC73 from Guadeloupe (See also Figure 4).

Region	Xa-FJ1		GPE PC73		Variation Type (Reference = Xa-FJ1)	Fragment Length (nt)		Predicted Cause of Variation	Affected Gene(s)
	Begins	Ends	Begins	Ends		Xa-FJ1	GPE PC73		
R1	219,400	219,400	219,691	271,791	Insertion	0	52,101	Prophage integration	XALc_0171-XALc_0242 (72 specific genes in GPE PC73)
R2	679,518	681,202	731,933	731,933	Deletion	1685	0	Recombination/assembly defect	XaFJ1_GM000644; XaFJ1_GM000645; XaFJ1_GM000646
R3	1,165,743	1,168,928	1,216,465	1,216,465	Deletion	3186	0	Recombination/assembly defect	XaFJ1_GM001035; XALc_1056
R4	1,727,177	1,727,177	1,774,582	1,780,815	Insertion	0	6234	Recombination/assembly defect	XaFJ1_GM001510; XALc_1529
R5	1,736,246	1,736,755	1,789,885	1,790,986	Complex Indel	510	1102	Prophage integration	XALC_1536-XALC_1545 (10 specific genes in GPE PC73); XaFJ1_GM001517-XaFJ1_GM001525 (9 specific genes in Xa-FJ1)
R6	1,736,873	1,741,929	1,791,101	1,795,842	Complex Indel	5057	4742	Prophage integration	
R7	1,742,065	1,742,065	1,795,979	1,796,122	Insertion	0	144	Prophage integration	
R8	1,742,742	1,742,742	1,796,839	1,797,116	Insertion	0	278	Prophage integration	
R9	1,778,246	1,784,662	1,832,620	1,832,620	Deletion	6417	0	Recombination/assembly defect	XaFJ1_GM001532; XALc_1551
R10	2,484,556	2,484,556	2,532,506	2,535,350	Insertion	0	2845	Recombination/assembly defect	XaFJ1_GM002152; XALc_2151; XALc_2152
R11	26,75,326	2,679,992	2,726,120	2,726,120	Deletion	4667	0	Transposable elements	XaFJ1_GM002293; XaFJ1_GM002292; XaFJ1_GM002291; XALc_2290
R12	3,041,680	3,041,680	3,087,815	3,088,684	Insertion	0	870	Transposable elements	XALc_2603; XALc_2604
R13	3,377,378	3,377,378	3,424,367	3,424,696	Insertion	0	330	CRISPR-Cas	Intergenic region which contains clustered regulatory interspaced short palindromic repeats
R14	3,377,647	3,377,713	3,424,965	3,424,965	Deletion	67	0	CRISPR-Cas	
R15	3,377,912	3,377,912	3,425,163	3,425,227	Insertion	0	65	CRISPR-Cas	
R16	3,460,518	3,463,513	3,507,852	3,507,852	Deletion	2996	0	Transposable elements	XaFJ1_GM002988; XaFJ1_GM002989; XaFJ1_GM002990; XALc_2969

**Table 3:** Putative function of the 10 genes of the chromosome of *X. albilineans* strain Xa-FJ1 from China that were not present in the genome of strain GPE PC73 from Guadeloupe.

Specific Gene	Annotation	Location
XaFJ1_GM001517	zona occludens toxin	the plasticity zone, R5-R8
XaFJ1_GM001518	hypothetical protein	the plasticity zone, R5-R8
XaFJ1_GM001519	hypothetical protein	the plasticity zone, R5-R8
XaFJ1_GM001520	hypothetical protein	the plasticity zone, R5-R8
XaFJ1_GM001521	hypothetical protein	the plasticity zone, R5-R8
XaFJ1_GM001522	hypothetical protein	the plasticity zone, R5-R8
XaFJ1_GM001523	DNA-binding protein	the plasticity zone, R5-R8
XaFJ1_GM001524	replication initiation protein	the plasticity zone, R5-R8
XaFJ1_GM001525	hypothetical protein	the plasticity zone, R5-R8
XaFJ1_GM002989	transposase	transposable elements, R16

## Supplementary Materials

The following are available online at https://www.mdpi.com/2076-2607/8/2/182/s1, Figure S1: Genome organization and gene distribution in strain Xa-FJ1 of *X. albilineans* from China. (A) Chromosome, and (B) Plasmid. From the outside to the inner circles: Coordinates (MB), protein-coding genes, COG annotations, KEGG annotations, GO annotations, ncRNA genes, percent GC content and GC skew. The outer part of the second, third, fourth, and fifth circle illustrates the forward strand, and the inner parts illustrate the reverse strand of the genome. Different colors of gene annotations refer to different categories summarized in the legend with colored squares (from the left to the right column: COG, KEGG, GO, and ncRNA). The red color of the GC content line plot indicates that GC percentage is higher than average while the green color of the same line plot indicates that GC percentage is lower than average. The purple and green inner circle shows the GC skew (G−C)/(G + C) using a 3724 bp window in Figure S1A and a 500 bp window in Figure S1B. Figure S2: GO annotation of the coding sequences of the genome of *X. albilineans* strain Xa-FJ1. Among the 24 subcategories of biological processes of the GO database, the largest category of Xa-FJ1 was assigned to the metabolic process (1186 genes). Figure S3: KEGG annotation of the coding sequences of the genome of *X. albilineans* strain Xa-FJ1. A total of 1491 genes of 2987 annotated genes were involved in metabolism, especially the metabolic pathways belonging to global and overview maps (529 genes). Figure S4: COG functional classification of the coding sequences of the genome of *X. albilineans* strain Xa-FJ1. Amino acid transport and metabolism was ranked the third largest term among the 25 classes of functional categories of the COG database. Figure S5: Comparison of the spacer distribution in the CRISPR-1 and CRISPR-2 systems between *X. albilineans* strains GPE PC73 and Xa-FJ1. Each box represents a CRISPR spacer and the spacer positions were numbered inside each box from the trailer end spacer (number 1) to the leader end spacer (as reported by Pieretti et al. [11]). Meaning of the colored boxes: blue = spacer identical between the two strains; orange and grey = sequence of spacer different between the two strains; yellow, green and purple = presence of additional spacer(s). R13-R15 refer to InDels described in the text. Table S1: Characteristics of 16 strains of *X. albilineans* from worldwide locations and one strain of *X. pseudalbilineans* used in this study. Table S2: General features of the plasmid of strain Xa-FJ1 of *X. albilineans* from China compared to the three plasmids of strain GPE PC73 from Guadeloupe. Table S3: Summary of gene annotation of strain Xa-FJ1. Table S4: Virulence factors retrieved from the VFDB database. Table S5: Putative function of 82 genes present in the chromosome of strain GPE PC73 of *X. albilineans* from Guadeloupe and absent in strain Xa-FJ1 from China. Table S6: Putative function of the genes identified in plasmids PlasmI and PlasmIII of strain GPE PC73 of *X. albilineans* from Guadeloupe.
